# Chemical Characterization, Cytotoxic Activity, and Antimicrobial Potential of Nanoemulsions Based on *Cymbopogon citratus* Essential Oils

**DOI:** 10.1021/acsomega.5c06077

**Published:** 2025-07-23

**Authors:** Serpil Demirci Kayiran, Umay Merve Guven Bolgen, Tilbe Cevikelli, Aylin Balcı Ozyurt, Esra Eroglu Ozkan, Elif Ferahoglu, Saliha Kırıcı, Esmeray Kuley, Yesim Ozogul, Fatih Ozogul

**Affiliations:** † Faculty of Pharmacy, Department of Pharmaceutical Botany, 37506 Cukurova University, 01330 Adana, Turkey; ‡ Faculty of Pharmacy, Department of Pharmaceutical Technology, Cukurova University, 01330 Adana, Turkey; § Department of Pharmaceutical Technology, Faculty of Pharmacy, Bahçeşehir University İstanbul, 34349 İstanbul, Turkey; ∥ Department of Pharmaceutical Toxicology, Faculty of Pharmacy, Bahçeşehir University İstanbul, 34349 İstanbul, Turkey; ⊥ Department of Pharmacognosy, Faculty of Pharmacy, İstanbul University, 34452 İstanbul, Turkey; # Department of Field Crops, Faculty of Agriculture, Cukurova University, 01330 Adana, Turkey; ¶ Department of Fishing and Fish Processing Technology, Faculty of Fisheries, University of Cukurova, Balcali, 01330 Adana, Turkey; ∇ Biotechnology Research and Application Center, Cukurova University, 01330 Adana, Turkey

## Abstract

The essential oils (EOs) of *Cymbopogon citratus* (DC.) Stapf are globally recognized for their antimicrobial properties. However, their therapeutic potential is limited by their low solubility and potency. This study aimed to investigate the chemical constituents, cytotoxic activity, and antimicrobial potential of nanoemulsions based on *C. citratus* EOs. Thirteen primary components were analyzed using gas chromatography–mass spectrometry. The major components were geraniol acetate (50.81%), citral (16.97%), β-citral (12.95%), and geranial (9.45%). Nanoemulsions containing three different concentrations (1%, 5%, and 10%) of EOs were evaluated regarding the droplet size, polydispersity index, pH, conductivity, and viscosity. The results demonstrated that nanoemulsions with spherical shapes and droplet sizes less than 143 nm were successfully formed at all tested concentrations, with average sizes of 143, 132, and 82 nm, respectively. Furthermore, all formulations were physically stable, even after undergoing stability testing. The EO of *C. citratus* exhibited potent antibacterial activity against a broad range of foodborne pathogens, including both Gram-positive and Gram-negative bacteria, with *Salmonella Paratyphi* A being the most susceptible strain. The aforementioned nanoemulsions were more effective against methicillin-resistant *Staphylococcus aureus* and *Bacillus cereus*. The nanoemulsions did not induce any significant cytotoxicity within the concentration range of 0.05–2 μg/mL in 24 h (*p* > 0.05). The cell viability consistently remained above 85%, even at the highest concentration tested. These findings suggested the strong potential of *C. citratus* EOs and their nanoemulsion forms as natural antimicrobial agents for applications in the food and pharmaceutical industries.

## Graphical abstract

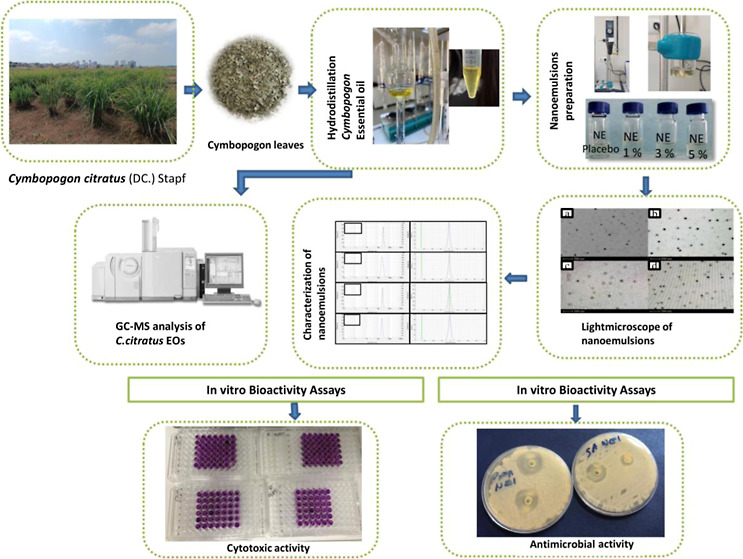

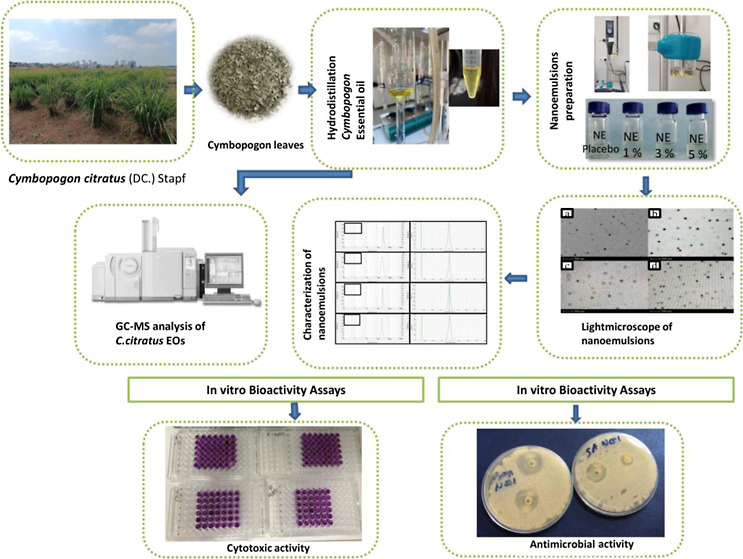

## 1 Introduction

Essential oils (EOs) are natural volatile compounds extracted from plants and are widely used across various industries, particularly in pharmaceuticals and food. In the pharmaceutical sector, EOs are used in medication formulations, aromatherapy, and alternative medicine because of their antibacterial, anti-inflammatory, antioxidant, and therapeutic properties.^1^ In the food sector, EOs are frequently used as natural flavorings and preservatives.^2,3^ Their antimicrobial and antioxidant properties help extend the shelf life of food products by inhibiting the growth of spoilage and pathogenic microorganisms.^4,5^

*C. citratus* (DC.) Stapf is an aromatic plant belonging to the Poaceae family, commonly known as lemongrass. Lemongrass grows naturally in India, Africa, South America, and Indonesia. It is used in the pharmaceutical industries for making cosmetics and perfumes and in the food industry as a culinary flavoring.^6,7^ The leaves, aerial parts, and roots of *C. citratus* are used in cosmetics and perfumes; in traditional Chinese medicine, they are known to possess antipyretic, analgesic, spasmodic, diuretic, and calming properties.^8^

The EOs of *C. citratus* have many chemical constituents such as citral, myrcene, geraniol, citronellol (cymbopogonol and cymbopogone), and α-oxobisabolene.^9,10^ Citral is the major compound in *C. citratus* EOs, with concentrations ranging from 30% to 90%. Citral is an aldehyde with two geometric isomers: neral (citronellal A) and geranial (citronellal B). One of the factors determining the quality of *C. citratus* EOs is its citral content.^10,11^ *C. citratus* EOs comprise phenolic compounds, flavonoids, and other phytochemicals having antibacterial, antifungal, anticancer, antioxidant, antinociceptive, antiobesity, antidiarrheal, and anti-inflammatory properties.^12–17^

Ghaly et al. reported that crude extracts of *C. citratus* partially inhibited the growth of some bacteria.^18^ Kawhena et al. found that *C. citratus* EO inhibited *Botrytis cinerea* and *Penicillium* spp., suggesting its use in edible coatings for pomegranate preservation.^19^ Prajapati et al. reported strong antifungal activity of the powder and EOs of *C. citratus* leaves against *Candida albicans*.^20^ Geraldi et al. also confirmed the antifungal activity of *C. citratus* extract against *C. albicans*.^21^ Sharma et al. also demonstrated its effectiveness against some fungi.^22^ Vargas et al. reported the antibacterial effects of *C. citratus* EOs against *Staphylococcus aureus* and *Escherichia coli*. In contrast, Luang-In et al. demonstrated its effect against *Cutibacterium acnes* and *Streptococcus agalactiae*.^23,24^ Thuy et al. showed the inhibitory effect of *C. citratus* EOs against SARS-CoV-2 using visual screening and simulation methods.^25^ Pan et al. investigated the antioxidant and anticancer activities of *C. citratus* EOs.^26^

Despite their numerous benefits, EOs have a fast evaporating nature, low stability, and poor solubility. Nanoemulsions have displayed strong potential for effectively stabilizing and delivering lipophilic compounds, including nutraceuticals, drugs, flavors, antioxidants, and antimicrobial agents. They serve as emulsifiers, stabilizing a lipid phase dispersed in a continuous aqueous phase. Nanoemulsions are highly stable toward gravitational separation and droplet aggregation due to their small droplet size; they also enhance the bioavailability of encapsulated compounds.^27,28^ They are prepared using various methods involving low and high energy. High-energy processes, such as emulsification-evaporation and solvent displacement, provide full control over the particle size, although they can result in chemical degradation and scalability challenges. In contrast, low-energy techniques are based on the physicochemical behavior of both surfactants and the oil phase; they are less energy-consuming and more manageable in terms of scalability.^29^

*C. citratus* EO-based nanoemulsions were prepared by Boudechicha et al.^30^ The formulation exhibited extremely high antibacterial activity against *Aspergillus flavus*, *Aspergillus parasiticus*, *Aspergillus carbonarius*, *Aspergillus ochraceus*, *Aspergillus oryzae*, *Penicillium verrucosum*, *P. chrysogenum*, *Fusarium graminearum*, *F. moniliforme*, and *F. oxysporum*. This study was conducted to chemically characterize the EOs of *C. citratus* and evaluate the cytotoxic and antimicrobial potential of its nanoemulsion formulations by assessing their physicochemical properties, stability, and efficacy against various microbial strains. The goal was to enhance the application of these nanoemulsions as natural antimicrobial agents in the food, cosmetic, and healthcare industries.

## 2 Materials and Methods

### 2.1 Plant Material

The seeds of *C. citratus* were obtained from India. The *C. citratus* samples for this study were procured from plants cultivated in the fields of the Agriculture Faculty, Cukurova University, Adana, Southern Turkey. The samples were identified by Demirci Kayiran. Voucher specimens were deposited in the Herbarium of the Faculty of Pharmacy, Cukurova University, Adana, Turkey.

### 2.2 Preparation of *C. citratus* EOs

#### 2.2.1 Hydrodistillation for EO

The plant material was allowed to dry in the shade at room temperature until a constant weight was achieved, thereby ensuring surface moisture removal without degrading the volatile compounds. A Clevenger apparatus (ISOLAB, Turkiye) was used to hydrodistilize 50 g of dried materials with 500 mL of distilled water for 3 h. The distillation was performed in triplicate under identical conditions, yielding an average of approximately 1.1 mL of EO per 100 g of plant material. The obtained EO was separated from the aqueous phase, dried over anhydrous sodium sulfate, and stored in sealed vials at 4 °C until further analysis. For gas chromatography–mass spectrometry (GC/MS) analysis, the EO was diluted with dichloromethane (CH_2_Cl_2_) in a ratio of 1:3 and directly injected into the GC/MS system.

### 2.3 GC–MS Analysis of *C. citratus* EOs

The *C. citratus* EOs were analyzed using a GC system (7890B, Agilent) coupled to a mass spectrometer (7010B, Agilent) equipped with an electron impact ionization source operating at 70 eV (Agilent Technologies Inc., CA, USA). GC separation was performed on a DB-WAX capillary column (60 m × 0.25 mm I.D., film thickness 0.25 μm; J&W Scientific, CA, USA). The injector and detector temperatures were maintained at 250 °C. Helium served as the carrier gas at a constant flow rate of 1.4 mL/min. The EO sample (1.0 μL) was injected in a split mode (20:1). The oven temperature was initially set at 40 °C for 4 min, increased to 250 °C at a rate of 5 °C/min, and then held for 10 min. The mass spectrometer was operated in full scan mode (*m*/*z* 40–400), with the ion source temperature at 230 °C and the quadrupole temperature at 150 °C. Compound identification was performed by comparing the mass spectra of the components with those in the NIS14L mass spectral library and calculating retention indices (RI), which were then compared with published values. The percentage composition of each compound was determined using NIS14L software based on peak area normalization.

### 2.4 Pseudoternary Phase Diagrams and Preparation of Nanoemulsion Formulations

NE formulations were prepared using the pseudoternary phase diagram method. The pseudoternary phase diagrams were developed using a Cremophor EL and Tween 80 mixture (1:1, w/w) as the surfactant system, ethanol as the cosurfactant, and distilled water as the aqueous phase. Cremophor EL and Tween 80 are nontoxic and biocompatible nonionic surfactants extensively applied in pharmaceutical preparations because of their emulsifying ability. Ethanol is a short-chain alcohol homologue acting as a cosurfactant to facilitate the reduction of interfacial tension and enhancement of interfacial film elasticity.^31^ Surfactant/cosurfactant (S/CoS or Smix) weight ratios of 1:1, 1.5:1, and 2:1 were examined, besides Cremophor and Tween 80 used as surfactants in a 1:1 ratio. The oil-to-Smix ratio (1:9, 2:8, 3:7, 4:6, 5:5, 6:4, 7:3, 8:2, and 9:1) was prepared from nine different concentrations. All preparations were titrated at room temperature with distilled water while being stirred (700 rpm) with a mechanical stirrer (Heidolph, Germany). Distilled water was added to the system until it clouded, and the volume was recorded. The pseudoternary phase diagrams were constructed using computer software to determine the percentage ranges of oil, Smix, and distilled water within which NE formation was possible.^32–34^ The formulation giving an NE system was selected based on the phase diagram results. The optimum NE ratio was identified at the center of the NE formation areas. The same low-energy method applied previously in preparing pseudoternary phase diagrams was used to prepare the formulation by titrating oil and Smix with distilled water at room temperature under mechanical stirring. *C. citratus* EO was added to the oil phase in amounts corresponding to final concentrations of 1%, 5%, and 10% (w/w) in the formulation. The placebo was obtained by preparing the formulation using the same method employed for *C. citratus* EO but in the absence of oil.^33,35^

### 2.5 Characterization of Nanoemulsion Formulations

#### 2.5.1 Zeta Potential, Droplet Size, and Droplet Sizedistribution

The zeta potential, droplet size, and droplet size distribution of placebo and EO-based NEs were analyzed using dynamic light scattering (Horiba SZ100, Japan). The mean droplet size (*Z*-average diameter) was measured, and the data were presented as mean diameter ± standard deviation (SD).

#### 2.5.2 Morphology of Nanoemulsions

The shape of the microemulsions was examined using a high-resolution inverted microscope (DMIL LED Fluo, Leica, Germany). The results are presented in the appropriate figures.

#### 2.5.3 pH Value

The pH of placebo and EO-based NE formulations was measured at 25 ± 1 °C using a Mettler Toledo FiveGo digital pH meter (Switzerland). The test was performed in triplicate, and the values were reported as the mean ± SD.

#### 2.5.4 Conductivity

The electrical conductivity of placebo and EO-based NEs was measured using a Mettler Toledo FiveEasy Plus conductivity meter (Switzerland). The samples were held at 25 °C ± 1 °C. The measurements were performed in triplicate and presented as mean ± SD.

#### 2.5.5 Rheological Properties and Viscosity

A Brookfield cone and plate rheometer was used to measure the shear stress, shear rate, and apparent viscosity of placebo and EO-based NEs in triplicate (Brookfield DV3THACJ0; Brookfield Engineering Laboratories., Inc., MA, USA) under controlled-temperature conditions (25 ± 0.5 °C). The rotation speed was adjusted to 10–100 rpm.

### 2.6 Stability of Nanoemulsion Formulations

The thermodynamic stability assays were performed to elucidate the stability of the formulations in the long term. The formulations were analyzed by using centrifugation, freeze–thaw, and heat–cool cycles. All formulations were centrifuged at 7000 rpm for 30 min. Subsequently, the formulations were subjected to a heat–cool cycle and then stored at 4 and 45 °C for 48 h. Then, a freeze–thaw cycle was performed. The formulations were transferred to vials and stored at −4 °C for 12 h and at room temperature for 12 h. Each experiment was conducted in triplicate.

### 2.7 Cytotoxic Activity

#### 2.7.1 Cell Line and Culture Conditions

This study used L929 (NCTC clone 929, CCL-1) murine fibroblast cells (ATCC), which are widely recognized for their application in dermal toxicity testing and evaluation of topical pharmaceutical formulations. The cells were maintained in high-glucose Dulbecco’s modified Eagle’s medium enriched with 10% fetal bovine serum, 1% nonessential amino acids, and 1% penicillin–streptomycin. The cultures were incubated under standard conditions (37 °C, 5% CO_2_). On reaching approximately 90% confluence, the cells were subcultured using a trypsin-ethylenediamine tetraacetic acid (EDTA) solution.

#### 2.7.2 Assessment of Cell Viability

The metabolic activity of cells following treatment was assessed using the 3-(4,5-dimethylthiazol-2-yl)-2,5-diphenyltetrazolium bromide (MTT) assay, which is a well-established colorimetric method for determining cell viability, proliferation, and cytotoxicity. The assay is based on the reduction of the yellow tetrazolium salt MTT to purple formazan crystals by mitochondrial dehydrogenase enzymes, which indicates viable cell activity. After incubation with the test substance, the resulting formazan was solubilized, and the absorbance was measured at 570 nm using a spectrophotometer. The experimental groups included NE1.5:1 placebo and NE1.5:1 formulations at concentrations of 1%, 5%, and 10%. The potential dose-dependent cytotoxicity was evaluated by exposing the cells to seven concentrations (0, 0.05, 0.1, 0.25, 0.5, 1, and 2 μg/mL) of the test substance for 24 h (*n* = 6 per group).

### 2.8 Antimicrobial Activity

#### 2.8.1 Bacterial Strains Used

Two fish spoilage bacteria, *Vibrio vulnificus* and *Proteus mirabilis*, were isolated from spoiled meat of sardine, mackerel, and anchovy. The reference strains were obtained from recognized culture collections. Gram-negative *Salmonella Paratyphi* A (NCTC 13) was sourced from the National Collection of Type Cultures (London, United Kingdom). Gram-positive methicillin-resistant *S. aureus* (MRSA, ATCC 33591) and *Bacillus cereus* (ATCC 10876) were acquired from the American Type Culture Collection (MD, USA).^36^

#### 2.8.2 Agar Well Diffusion Method

The in vitro antibacterial properties of pure EOs and their nanoemulsion forms were determined using Mueller–Hinton agar (MHA, Merck 1.05437, Darmstadt, Germany), as described by Hwanhlem et al.^37^ Food-related bacteria were cultivated at 37 °C in nutrient broth (Merck 1.05443, Darmstadt, Germany) for 24 h and arranged to 0.5 McFarland cell density (108 colony-forming unit/milliliter (cfu/mL)). Each 100 μL bacterial cell culture was injected into a Petri plate containing 20 mL of MHA.

Five 5 mm wells were created in a solid medium. Four wells were inoculated with 50 μL of pure EOs or their nanoemulsion forms. As a control, Tween 80, Cremophor, or oleic acid was added to the other well. Then, Petri dishes were kept at 37 °C for 24 h for incubation. Following incubation, calipers were used to quantify the inhibition zones developed around each well in millimeters.

#### 2.8.3 Minimum Inhibitory and Bactericidal Concentrations

The CLSI microdilution method was used to calculate the minimum bactericidal concentration (MBC) and minimum inhibitory concentration (MIC).^38^ The bacteria were standardized to 0.5 MacFarland cell density after being cultured at 37 °C for 24 h. Mueller–Hinton broth (Basingstoke, UK) served as the medium. Each stock solution (50 mg/mL) was diluted and arranged to 0.19 μg/mL. The other tubes with MHB included bacteria and diluted stock solutions, whereas the control tube contained only pure culture or stock solution. After repeated preparation, the test tubes were incubated at 37 °C for 24 h. The bacterial growth in the test tubes was compared to that in the control tubes, and the MIC of the tubes with the least degree of bacterial growth suppression was recorded. Based on the MIC results, the tubes free of bacterial growth were injected onto the surface of MHA. The MBC values were recorded in the Petri plates following a 24 h incubation period at 37 °C.

### 2.9 Statistical Analysis

Statistical evaluations were performed using GraphPad Prism software (version 10.2.3). The Shapiro–Wilk test was used to determine whether the data distribution was normal. Tukey’s multiple comparison test was then performed after a one-way analysis of variance. A *p* value less than 0.05 indicated a statistically significant difference.

## 3 Results and Discussion

### 3.1 Chemical Composition of EOs

The EO yield of *C. citratus* was determined as 1.1%. Previous studies reported variable yields ranging from 0.25% to 1.46% depending on distillation conditions.^39,40^ For instance, the EO yields obtained from dried leaves within 30–90 min ranged between 0.75% and 1.37%. Similarly, Variyana et al. reported a yield of 1.17% with a material-to-solvent ratio of 0.1.^40^

The GC–MS analysis revealed that the major constituents of *C. citratus* EOs were geranyl acetate (50.81%), citral (16.97%), β-citral (12.95%), geraniol (9.45%), and linalool (1.70%) (Table 1). These components accounted for approximately 85% of the total oil content, highlighting their potential contribution to the biological activity of the oil. However, the predominance of geranyl acetate at such a high concentration was unusual. Structurally similar compounds such as geraniol (9.45%), nerol (0.30%), and nerol acetate (3.62%) were also detected, raising the possibility of coelution and spectral overlap. Compound identification was solely based on mass spectral matching with the NIST library, without confirmation through Kovats RI or authentic standards. Therefore, the precise quantification of geranyl acetate might be limited in accuracy, representing a limitation of the study.^41^

**Table 1:** Chemical Constituents of *C. citratus* EOs^a^

no.	component	RT (min)	relative peak area (%)	retention index (RI)
1	β-myrcene	17.53853	0.47	1189.86
2	5-hepten-2-one, 6-methyl-	25.49545	0.48	1364.08
3	1-octen-3-ol	30.16152	0.40	1476.45
4	trans-chrysanthemal	32.81685	0.27	1476.45
5	linalool	34.28112	1.70	1539.39
6	isogeranial	35.52128	0.75	1602.04
7	β-citral	40.11048	12.95	1712.94
8	nerol acetate	41.73738	3.62	1759.16
9	citral	41.89479	16.97	1763.63
10	geranyl acetate	42.83937	50.81	1790.47
11	nerol	43.94748	0.30	1825.08
12	geraniol	45.32267	9.45	1869.73
**total**			**98.17**	

a RT, retention time.

Literature reports varying compositions of major compounds in *C. citratus* EOs depending on geographical origin, plant part, and extraction methods. Geraniol has been reported as a major component in leaf-derived EOs (up to 53.1%);^42^ however, monoterpene aldehydes—geranial and neral—typically dominate, often accounting for 75%–80% of the oil.^43^ For instance, studies from Indonesia,^44^ Türkiye,^45^ Mexico,^46^ Iran,^47^ Algeria,^48^ Egypt,^49^ Brazil,^50,51^ and Thailand^24^ have reported *E*-citral (geranial) and *Z*-citral (neral) as the most abundant compounds, alongside β-myrcene, geraniol, and minor amounts of geranyl acetate.

According to the International Organization for Standardization (ISO standards), high-quality *C. citratus* EOs should contain at least 75% citral, composed of 35%–47% geranial and 25%–35% neral.^52^ The acceptable limits for other components include 1.5%–8% geraniol and 0.5%–6% geranyl acetate. In this study, the geraniol content was consistent with these standards; however, the geranyl acetate content significantly exceeded the expected range.

While geranyl acetate is not typically reported as a major constituent in *Cymbopogon citratus* EO, several studies have demonstrated that its concentration can vary significantly depending on the chemotype, geographic origin, harvest time, and extraction method. For instance, Plata-Rueda et al. (2020) identified geranyl acetate at 12.4% in *C. citratus* oil, following neral (24.6%) and citral (18.7%).^53^ Similarly, Arputha Bibiana et al. (2012) reported a geranyl acetate content of 15.47% in hydrodistilled oil, where geraniol was the dominant component (76.89%).^54^

A more comprehensive data set from a statistical analysis of 45 *Cymbopogon* EO samples, including lemongrass and citronella types, supports the potential for high geranyl acetate levels in certain cases. In this study, the compound was found in a broad range of concentrations, with the majority of samples containing less than 5%, but some outliers showing abundances as high as 70.784% (sample 38), 56.809% (sample 3), and 12.7225% (sample 27).^55^ This variability strongly suggests the existence of rare chemotypes in which geranyl acetate may become a major component.

In the present study, the relatively high content of geranyl acetate observed in the EO composition may reflect such chemotypic diversity. This variability may be attributed to factors including plant age, genetic variation, harvest time, postharvest treatment, and specific extraction method used.^44,56^ Therefore, although the current findings reflect the composition of the analyzed sample, variations in EO profiles across different studies emphasize the importance of comprehensive analytical validation.^57^

### 3.2 Pseudoternary Phase Diagrams and Preparation of Nanoemulsion Formulations

The region where the product of optimum NE stays in the surfactant ratios used was determined using pseudoternary phase diagrams. These diagrams help calculate the appropriate surfactant concentration to be added to the formulation.^58^ Therefore, a pseudoternary phase diagram was applied to find the optimal composition ratios of surfactant, cosurfactant (Smix), and oil. The surfactant–cosurfactant mixture was prepared using Cremophor EL/Tween 80 and ethanol in a ratio of 1:9–9:1. Cremophor EL (polyoxyl 35 castor oil) is used extensively for nanoemulsion formulations due to its superior emulsifying capability. These surfactants form highly stable oil-in-water nanoemulsions and nanoparticles because they are nonionic.^59,60^ Tween 80 was chosen for this study due to its polyoxyethylene content and long hydrophilic head groups causing steric repulsion, which is a fundamental factor in stabilizing NE droplets.^61^ Li et al. developed formulations containing mixed surfactants and had better emulsifying effects than single surfactants.^60^ Zeng et al. prepared a nanoemulsion containing Cremophor EL at a lower surfactant concentration. Short-chain alcohols were added as cosurfactants, minimizing the droplet size and increasing emulsion stability in these systems.^31^ In our study, Cremophor EL was used as the surfactant to enhance the solubilization of EOs; it contributed to the improved solubility of its active constituents.^31^ We used Cremophor EL and Tween 80 in combination as surfactants. Microemulsion systems formulated with a combination of Cremophor EL and Tween 80 provide smaller particle sizes and improved physical stability compared to those prepared with individual surfactants. The combined use of Cremophor EL and Tween 80 in microemulsion systems enhances the physical stability and reduces the potential toxicity. It allows for the formation of effective emulsions at lower total surfactant concentrations, which is particularly critical for minimizing the risk of toxicity.^62,63^

The NE regions of a diagram for each of the three Smix ratios were found. Their corresponding compositions at the center point of the NE regions of the corresponding diagrams are provided in Table 2. The surfactants with a mass ratio of 1.5:1 were Cremophor EL and Tween 80. The oil phase was oleic acid, and the cosurfactant was ethanol.

**Table 2:** Pseudoternary Phase Diagrams of the Compositional Ratios of the Blank Nanoemulsion Formulations and the Relative Nanoemulsion Areas

formulation (weight %)			
	NE1:1	NE1.5:1	NE 2:1
oleic acid	15.166	15.316	15.700
Cremophor EL	18.590	23.326	26.374
Tween 80	9.295	11.663	13.187
ethanol	27.886	23.327	19.780
water	29.063	26.366	24.958
NE area	404.051	412.595	397.502

Figure 1 shows the NE formation region in the pseudoternary phase diagram. A striking order of the NE formation region was observed where the Smix ratio was in the order 1.5:1 > 1:1 > 2:1. The NE formation region had a larger area with an Smix ratio of 1.5:1, confirming that NE formation was optimal at an Smix ratio of 1.5:1. Hence, this formulation was selected for characterization and activity assays.

**Figure 1:**
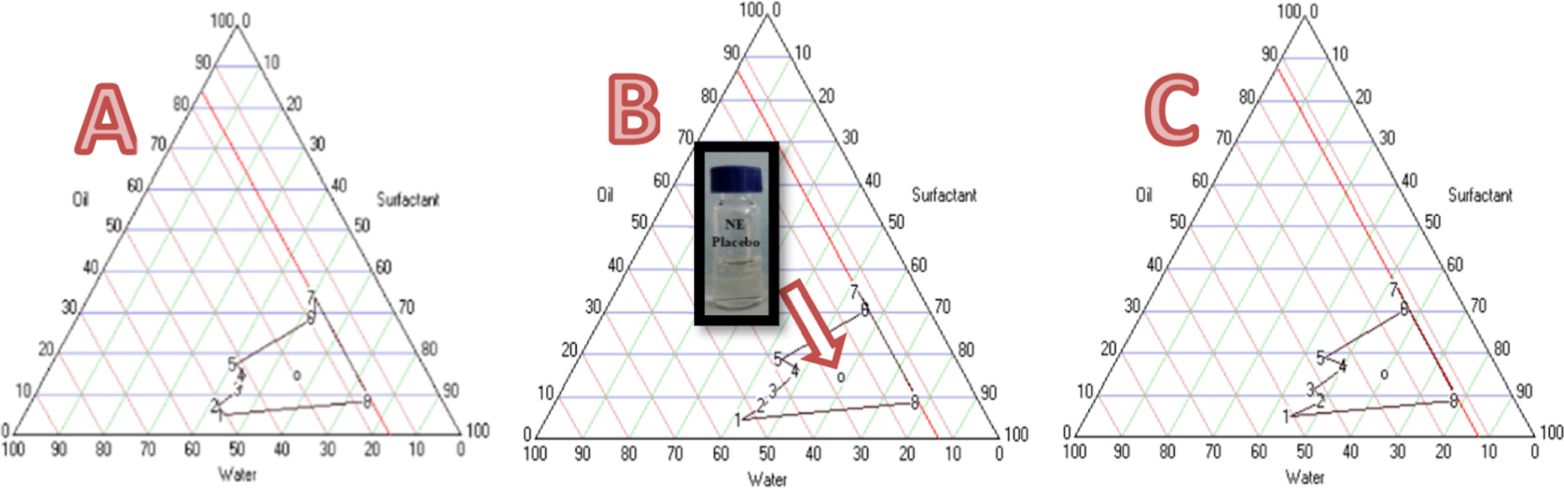
Pseudoternary phase diagrams of nanoemulsions (a: NE1:1; b: NE1.5:1; c: NE 2:1).

### 3.3 Characterization of Nanoemulsion Formulations

*C. citratus* EOs are highly unstable and volatile and prone to oxidation under standard conditions. Hence, they must be formulated with NEs for the better pharmacokinetic profile. The oil phase was prepared by adding 1%, 5%, and 10% (w/w) EOs of *C. citratus*. The best NEs exhibited a yellowish color with opacity against a blue background and the peculiar aromatic odor of *C. citratus* EO. The characterization properties of the formulation were examined according to the droplet size and distribution, pH value, conductivity, and viscosity.

#### 3.3.1 Zeta Potential, Droplet Size, and Droplet Size Distribution

The zeta potential measurements were performed by using a zetasizer, and the results are presented in Table 3. No significant differences were observed between the placebo and nanoemulsions containing three different concentrations of EOs, with all zeta potential values found to be close to zero in the negative range. Zeta potential is a key parameter for evaluating the physical stability of nanoemulsions. Generally, absolute zeta potential values above ±30 mV indicate strong electrostatic repulsion and stability in the system, particularly in emulsions formulated with ionic surfactants.

**Table 3:** Characterization of Nanoemulsions^a^

characterization	NE1.5:1 (Placebo)	NE1.5:1 (1%)	NE1.5:1 (5%)	NE1.5:1 (10%)
droplet size (nm ± SD)	150.533 ± 21.623	143.400 ± 20.881	132.267 ± 32.100	82.567 ± 14.261
zeta potential (mV ± SD)	–0.86 ± 0.04	–1.24 ± 0.18	–1.42 ± 0.016	–1.30 ± 0.22
PDI	0.485 ± 0.010	0.544 ± 0.075	0.520 ± 0.156	0.525 ± 0.148
pH	5.413 ± 0.047	5.557 ± 0.118	5.693 ± 0.040	6.043 ± 0.021
conductivity (μSm/cm ± SD)	17.207 ± 0.110	17.720 ± 0.036	22.250 ± 0.149	22.863 ± 0.093

a All values are presented as the mean ± SD from three independent measurements.

The zeta potential of nanoemulsions prepared using nonionic surfactants such as Cremophor EL and Tween 80 may typically be close to zero, although it can also exhibit higher absolute values depending on the composition of other formulation components and the concentration of the surfactants. In the study by Ashagrie et al. (2025), nanoemulsions containing *Caesalpinia decapetala* seed oil—a type of fixed (nonvolatile) oil—were formulated using nonionic surfactants such as Tween 20 and Tween 80.^64^ The emulsions were prepared with relatively low surfactant concentrations, approximately 1% w/w. Despite the use of nonionic surfactants, the nanoemulsions exhibited notably high zeta potential values, ranging from −32 mV to −58 mV, indicating strong electrostatic stabilization.^64^

Although a high absolute value of zeta potential is generally considered an indicator of electrostatic stability, in cases where the zeta potential is close to zero, this does not imply instability because these systems are stabilized by steric rather than electrostatic mechanisms. In such cases, adsorbed nonionic surfactant layers create a physical barrier that prevents droplet coalescence. In the study by Li et al. (2024), the zeta potential of the corosolic acid nanoemulsion was found to be almost zero, while fine physical stability, a narrow particle size distribution, and uniform dispersion were observed. This suggests that the enhanced steric stabilizers provided by the nonionic surfactants (Cremophor EL and Tween 80) and the cosurfactant ethanol are effective in inhibiting short-range attraction and coalescence, even though the electrostatic repulsion is not strong enough.^60^

The size distribution of NEs is another essential factor influencing properties, such as stability, functionalities in application processes, and bioavailability of the encapsulated active ingredients. The average droplet size, droplet size distribution, and polydispersity index (PDI) are indicators of the homogeneity of the dispersion. The size of the droplets can also significantly impact the stability, viscosity, and optical properties of the emulsions. NEs are visible, yellowish, and transparent at a temperature of 25 °C. The PDI value between zero and one reflects the uniformity and stability of the droplet size distribution in emulsions. A small value of PDI indicates that the droplets are closely packed, and the distribution of the nanoemulsion formulations is homogeneous. A PDI value of ≤0.7 corresponds to a monodisperse formulation, indicating that the nanoemulsion is homogeneous.^65,66^

A PDI value less than 0.25 indicates a narrow size distribution in the system; all other results (PDI values) show formulations within acceptable ranges.^32,67,68^ The optimum droplet sizes and PDI values of formulations are shown in Table 3. The average droplet size for the placebo, 1%, 5%, and 10% NEs was 150.533, 143.400, 132.267, and 82.567 nm, respectively. All formulations led to nanometer-scale results with the desirable traits. Furthermore, integrating EOs into the system caused a decrease in the droplet size. In addition, the droplet size decreased with an increase in the amount of EOs. De Silva et al. used Tween 80 as a surfactant to develop NEs loaded with EOs from *C. flexuosus*. The droplet size of the blank formulation was significantly larger (135.9 ± 3.89 nm) than the droplet size of NE containing EOs (78.46 ± 0.51 nm).^1^

#### 3.3.2 Morphology of Nanoemulsions

The surface morphology of the microemulsion formulations was characterized using high-resolution microscopy. Microscopy is a direct imaging approach used for observing nanoemulsions and assessing essential characteristics such as size, morphology, and aggregation state.^66^ The microscopic images of the NEs revealed the presence of globular droplets with well-defined outlines (Figure 2). The nanodroplet sizes were consistent with the droplet size measurements, confirming the accuracy of the size analysis.^69,70^ Similar behavior was observed in nanoemulsions prepared with EOs employing microscopic imaging techniques.^71,72^

**Figure 2:**
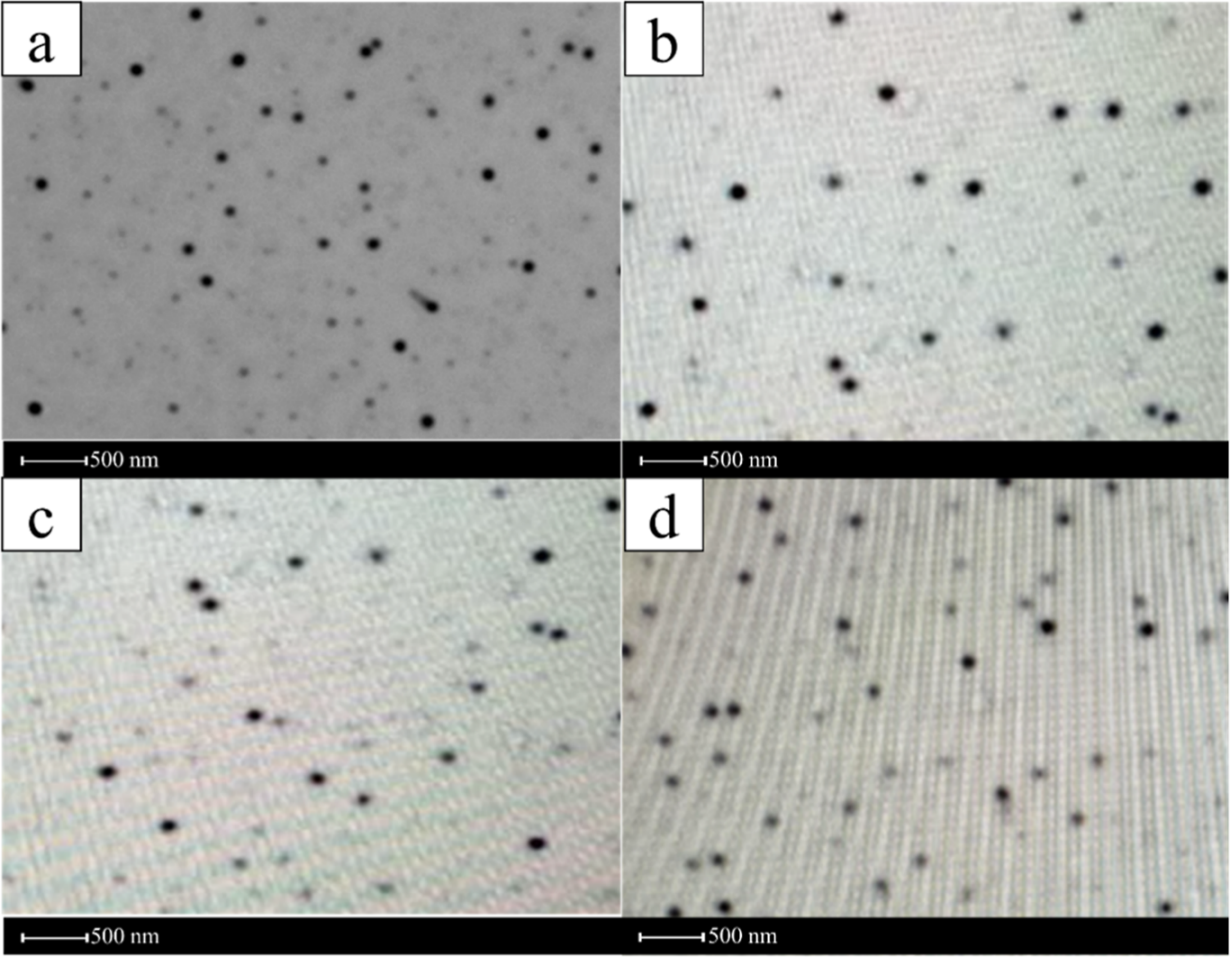
Morphological analysis of nanoemulsion formulations using high-resolution microscopy [a: NE1.5:1 (Placebo); b: NE1.5:1 (1% EO); c: NE1.5:1 (5% EO); d: NE1.5:1 (10% EO)].

#### 3.3.3 pH Value

The pH values were in the range 5.0–6.0 (Table 3). Each formulation had a neutral pH value, demonstrating nonacidic and nonirritant properties. The formulation of a nanoemulsion plays a vital role in its pH responsiveness and depends on the oil and surfactant type used for synthesizing the nanoemulsions. The maximum stability of nanoemulsions of lemon oil and sucrose monopalmitate, buffered with cosolvents (glycerol and propylene glycol) and a cosurfactant (lysolecithin), was between pH 5–6, with droplet growth and aggregation noted at lower or alkaline pH.^73^

#### 3.3.4 Conductivity

It is vital to determine the emulsion type using conductivity measurements. The results of the optimized formulations are presented in Table 3. Given that water-in-oil (w/o) emulsions typically exhibit lower conductivity, the observed result confirmed that the formulation was of the oil-in-water (o/w) type. This finding was consistent with the expected result, as the oil phase remained the dispersed internal phase within the continuous aqueous phase.^74^

#### 3.3.5 Viscosity and Rheological Properties

The viscosity of nanoemulsions is affected by various formulation components, including the oil phase, surfactant concentration, and temperature applied during preparation. Nanoemulsions are classified as low to medium viscous. Hence, a viscosity of 150–200 mPa s has been reported to be adequate for a slightly more viscous but still fluid nanoemulsion. Table 3 shows formulations with a viscosity range of 276.450 to 152.400 mPas, revealing Newtonian behavior. Therefore, the formulations in our study were within specified dosages with adequate flow properties and reasonable viscosity values.^75^

The rheograms presented in Figure 3 were used to evaluate the rheological behavior. It was concluded that the nanoemulsions displayed constant viscosity and a linear trend against the shear rate, indicating good stability and applicability in a pharmaceutical dosage form. The rheological behavior of the emulsions was predictable and reproducible, owing to the linear characteristic of the nanoemulsions, which has practical implications for both processing and application. Especially, its constant viscosity favorably enabled uniform and repeatable distribution over mucosal surfaces. Such uniformity facilitated practical considerations, for example, convenient and continuous packaging.^71,76^

**Figure 3:**
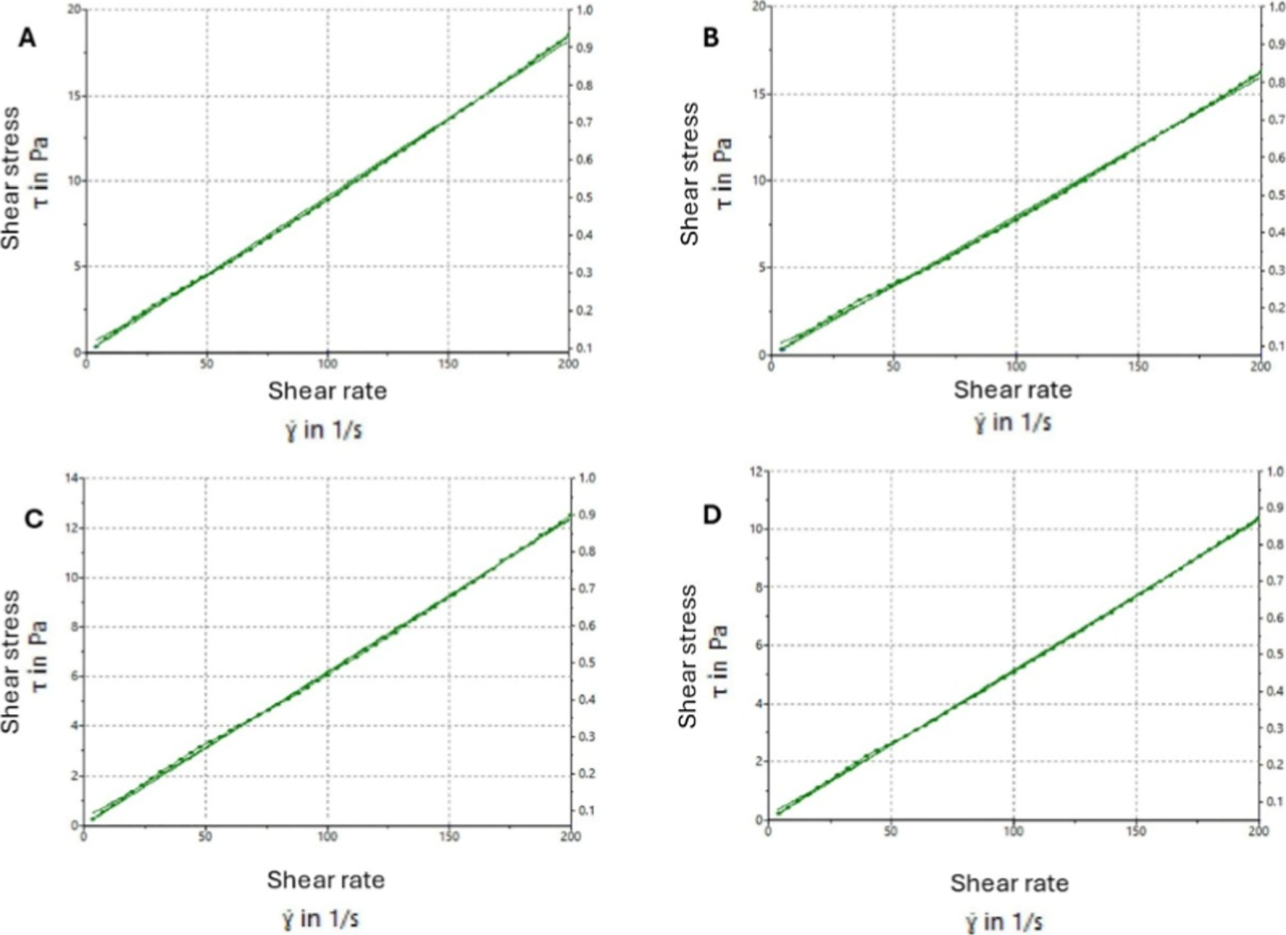
Rheograms showing the relationship between shear rate and shear stress. a: NE1.5:1 (Placebo); b: NE1.5:1 (1% EO); c: NE1.5:1 (5% EO); d: NE1.5:1 (10% EO).

### 3.4 Stability of Nanoemulsion Formulations

The formulations can undergo coalescence, creaming, cracking, precipitation, or phase separation over longer time scales. NE stability is a key parameter influencing their properties. This study aimed to accelerate possible changes during storage by centrifuging all NE formulations to identify factors potentially influencing their stability.^60,61^ Both placebo and EO formulations retained the same appearance and properties as those before the treatment. These findings showed no signs of precipitation, phase separation, or degradation during centrifugation, demonstrating the remarkable stability of NE formulations during centrifugation.^77^ Water at elevated temperatures can cleave hydrogen bonds in polyoxymethylene-based nonionic surfactants, leading to the loss of solubility. Thus, the system undergoes a transition from the translucent state to the turbid or delaminated state. The stability of this transient phenomenon becomes quite critical while examining the temperature stability of nanoemulsion systems with more than one active surfactant.^60^ In our study, heating–cooling and freeze–thaw tests were performed, revealing no noticeable change in the properties of the formulations.

### 3.5 Cytotoxic Activity

This study investigated the cytocompatibility and bioactivity of NE1.5:1 nanoemulsion formulations using the L929 murine fibroblast cell line and the MTT assay. As shown in Figure 4, none of the concentrations tested (0.05–2 μg/mL) resulted in statistically significant reductions in cell viability after 24 h (*p* > 0.05). All the tested formulations were found to be nontoxic because the cell survival was above 85% at the highest concentration of 2 μg/mL. Ortega et al. found no toxic effects of *Cymbopogon* EOs and their most essential components, citral and myrcene, on human keratinocytes and fibroblast cells after a 24 h exposure.^78^ Similarly, Salsabila et al. reported low cytotoxicity of lemongrass EOs exerted on NIH-3T3 fibroblast cells, demonstrating that the IC_50_ value surpassed 40 μg/mL, consistent with our findings.^79^ On the contrary, the concentration-dependent cytotoxic effects of *Cymbopogon* EOs were reported by other studies. For example, Bayala et al. and González et al. reported the cytotoxic activity of EOs from *C. citratus* and *Cymbopogon giganteus* in tumor cell lines and citral as a candidate antiproliferative compound. Such discrepancies can be ascribed to variations in the chemical constitution of the oil, the extraction method employed, the concentration tested, or the unique cell line used.^80,81^

**Figure 4:**
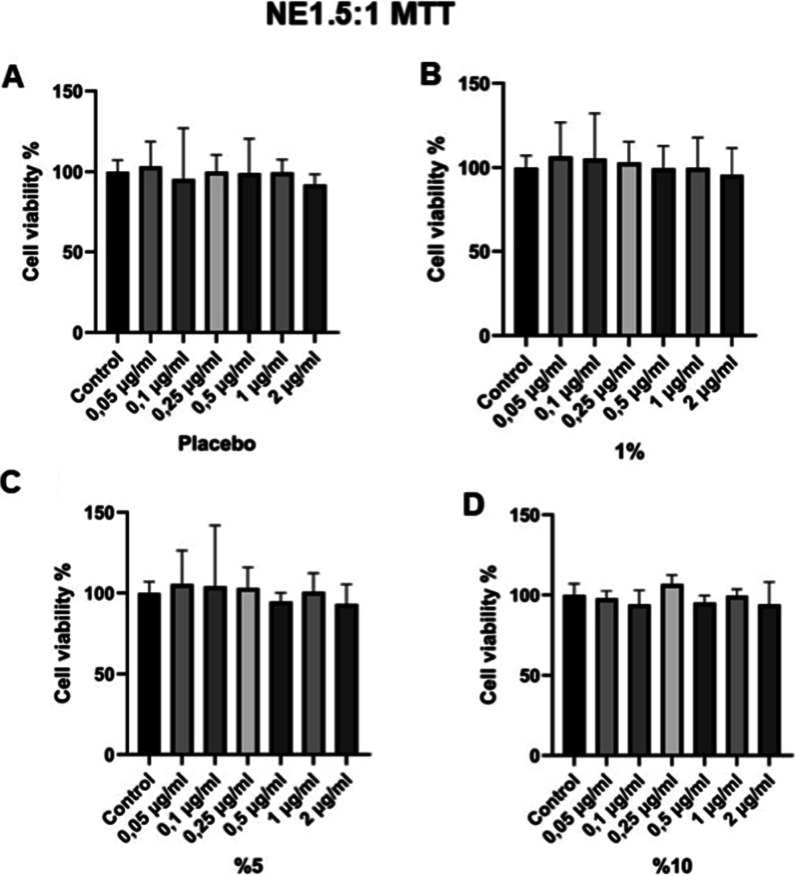
Cell survival in study groups.

These results were in agreement with several recent studies showing the biocompatibility of nanoemulsion-based systems prepared with nonionic surfactants, for example, Tween 80 and Cremophor EL. For instance, Khadem Nezhad and Zarei examined a nanoemulsion based on *Hypericum perforatum* and reported more than 90% viability in human gingival fibroblasts even at a concentration of 10 μg/mL.^82^ Similarly, Zhou et al. reported extremely low cytotoxicity of nanoemulsions based on various plant oils. The fibroblast cell viability (≥80%) was maintained even at the high nanoemulsion concentrations used, thus validating our results.^83^ The cytotoxicity data obtained showed that the NE1.5:1 nanoemulsion formulation exhibited a high degree of biocompatibility with fibroblast cells, indicating its promising safety profile for transdermal drug delivery. This was also supported by recently published studies demonstrating excellent skin tolerance and low irritancy of nanoemulsions produced with a combination of nonionic surfactants and natural oil phases. For instance, Verma et al. formulated a clove oil-based nanoemulsion. They evaluated its safety using in vitro fibroblast assays and ex vivo skin permeation tests, revealing no significant alteration in skin architecture or loss of viability.^84^ Similarly, Moghimi et al. examined the transdermally delivered diclofenac-loaded nanoemulsion and recorded more than 85% L929 cell viability besides improved skin penetration, supporting the safety and efficacy of the formulation.^85^ Moreover, Yousefi et al. indicated that oily nanoemulsions containing Tween 80 or Cremophor EL as surfactants caused minimal irritation and cytotoxicity in human fibroblast and keratinocyte models, corroborating our findings. Thus, the NE1.5:1 formulation may be used as a safe carrier for transdermal delivery and can be beneficial in pharmaceutical or cosmeceutical products requiring controlled topical release.^86^

### 3.6 Antimicrobial Activity

The inhibition zone of *C. citratus* EOs and their nanoemulsion forms on food-related bacteria is shown in Table 4. Pure EO showed the highest zone of inhibition for all bacteria tested, especially for the Gram-negative foodborne pathogen *S. Paratyphi* A (57.17 mm) (*p* < 0.05). The effect of *C. citratus* EO was statistically similar for MRSA and *P. mirabilis* (approximately 44 mm), whereas the lowest zone of inhibition was noted against *B. cereus* with 31.00 mm. However, Laassami et al.^48^ showed that *S. aureus* strains had the highest bacterial sensitivity for *C. citratus* EO among the studied microorganisms. Valková et al. found that the inhibition zones of *C. citratus* EO against Gram-positive and Gram-negative bacteria ranged from 2.67 mm for *S. marcescens* to 7.68 mm for *Y. enterocolitica* and 3.67 mm for *B. subtilis* to 8.33 mm for *M. luteus*.^87^ *S. aureus* (30.00 mm), *Streptococcus mutans* (38.00 mm), and *E. coli* (39.00 mm) were all significantly inhibited by *C. citratus* EO in vitro.^88^ Shendurse et al. found that *C. citratus* EO produced inhibition zones of 32.0, 48.0, and 21.0 mm against the three Gram-positive pathogens *S. aureus*, *B. subtilis*, and *B. cereus*, respectively.^89^ In contrast, an inhibition zone of 23.0 mm was reported against *Proteus vulgaris*, the only Gram-negative pathogenic bacteria; no inhibition zone was observed against *Pseudomonas aeruginosa* or *E. coli*. Saide et al. demonstrated that the *C. citratus* EO showed an average antibacterial activity of 17.33 mm against *E. coli* at a concentration of 100%.^90^ This effectiveness, however, dropped at lower concentrations: 50% (14.8 mm) and 75% (16.5 mm). A number of variables, including the size of the inoculum, the antimicrobial doses used, and the bacterial status (resistance, susceptibility, persistence, and tolerance), might influence the effectiveness of EOs at stopping bacterial growth.^91^

**Table 4:** Inhibition Zone of *C. citratus* EOs and Their Nanoemulsion Forms on Fish Spoilage Bacteria and Foodborne Pathogenic Bacteria

	MRSA	S. Paratyphi A	B. cereus	V. vulnificus	P. mirabilis
pure EO	44.25 ± 1.89^a^ ^bA^	57.17 ± 0.62^aA^	31.00 ± 2.00^dA^	35.75 ± 1.71^cA^	43.75 ± 1.50^bA^
NE	15.67 ± 0.29^cE^	13.33 ± 1.32^dD^	22.33 ± 0.58^aD^	20.00 ± 1.00^bC^	21.33 ± 0.58^abE^
NE1.5:1 (1% EO)	20.67 ± 0.29^cD^	18.67 ± 1.15^dC^	23.00 ± 0.50^abD^	21.67 ± 1.53^bcC^	23.67 ± 1.15^aD^
NE1.5:1 (5% EO)	26.67 ± 0.58^abC^	24.50 ± 0.50^cB^	26.50 ± 1.00^bcC^	25.50 ± 1.53^bcB^	28.67 ± 1.15^aC^
NE1.5:1 (10% EO)	41.67 ± 3.51^aB^	25.00 ± 1.00^cB^	28.33 ± 1.53^cB^	26.67 ± 0.58^cB^	37.33 ± 1.04^bB^
Tween 80	9.33 ± 0.58^cF^	5.50 ± 0.50^dF^	11.83 ± 0.29^aF^	9.58 ± 0.52^cE^	10.75 ± 0.25^bF^
Cremophor	13.50 ± 0.50^bE^	5.62 ± 0.34^eF^	17.33 ± 0.58^aE^	11.33 ± 0.58^cD^	10.17 ± 0.76^dF^
oleic acid	6.08 ± 0.58^bG^	6.25 ± 0.29^bE^	9.00 ± 0.29^aG^	6.58 ± 0.58^bF^	3.83 ± 0.14^cG^

a Mean value ± SD (*n* = 3). Different letters indicate significant differences (*p* < 0.05) between the treatment groups of each bacteria (A–D) in the row or between the bacteria of each group (a–c) in the same column.

The antimicrobial properties of *C. citratus* EO were associated with the synergistic action of multiple biologically active components.^87^ A wide range of antibacterial capabilities of the components of these oils, including geranial, neral, and geraniol, have been reported against both Gram-positive and Gram-negative bacteria and fungi.^43,48,87,92^ Furthermore, the breakdown of cell shape and cell wall integrity has been linked to the antibacterial effects of limonene and geranyl acetate.^93,94^ In the present study, the strong antimicrobial properties of *C. citratus* EO might be due to its high contents of geraniol acetate, citral, β-citral, and geraniol. Moreover, the minor components of *C. citratus* EO, such as limonene, linalool, and myrcene, significantly increased the effectiveness of the oil.^43,95^ The most sensitive bacteria to nanoemulsion application were *P. mirabilis* and MRSA, whereas the most resistant one was *S. Paratyphi* A. The control nanoemulsion treatment (NE group) exhibited an inhibition zone between 13.33 (*S. Paratyphi* A) and 22.33 (*B. cereus*) mm on bacteria. The high antimicrobial activity of nanoemulsion formulations might be attributed to the electrostatic interaction between the nanoemulsion and the microbial cell wall, which increased the concentration of EO or its constituent at the target site; the fusion of the nanoparticle (emulsion) with the cell membrane; the stability of EO constituents and their sustained release; or the interaction with the microbial cell membrane due to the large surface area and passive transport across the membrane.^96,97^ Additionally, compared with free EO, NE loaded with *C. citratus* EO demonstrated a notable 2- to 4-fold increase in cell wall permeability and intracellular enzyme leakage, indicating strong antibacterial action.^98^

Moreover, the inhibitory effect of nanoemulsions on bacteria increased with increasing the EO content. In other words, NE1.5:1 (10% EO) was the group with the highest antimicrobial activity after pure EO among the nanoemulsion groups tested. Gago et al. reported similar findings, indicating that the antibacterial activity of EO-based nanoemulsions against *E. coli* increased with an increase in EO concentrations. The NE1.5:1 (10% EO) group showed the highest antimicrobial activity against MRSA (41.67 mm), followed by that against *P. mirabilis* (37.33 mm). The inhibition zone in the NE1.5:1 (10% EO) group against other bacteria was statistically similar. The inhibition zones in the NE1.5:1 (1% EO) and NE1.5:1 (5% EO) groups against the test microorganisms ranged from 18.67 and 24.50 mm (*S. Paratyphi* A) to 23.67 and 28.67 mm (*P. mirabilis*), respectively.^99^ Compared with the antibacterial activity of pure *C. citratus* EO, the *C. citratus* EO-based nanoemulsions produced by Bonferoni et al., Balasubramanian et al., and Noorbakhsh et al. displayed an enhanced antimicrobial impact.^100–102^ Similarly, Hebishy et al. demonstrated poorer antibacterial effectiveness of free/nonemulsified *C. citratus* EO against all studied pathogens (*S. aureus*, *Listeria monocytogenes*, and *E. coli* O157:H7) with an inhibitory halo of 0.29, 0.23, and 0.12 mm, respectively.^103^ *C. citratus* EO-based nanoemulsions included 1%, 5%, or 10% of the EOs in this study. Therefore, they have lower concentrations of bioactive EO compounds. This might explain why the antibacterial activity of pure EO was stronger than that of the nanoemulsion form.^68^ Therefore, the bacteriostatic efficacy of the nanoemulsion system increased with the addition of EOs, taking into account the EO content of the nanoemulsions.

Tween 80, Cremophor, and oleic acid used in the nanoemulsion formulation generally exhibited low antibacterial activity against bacteria. *B. cereus* was the most sensitive of the tested bacteria with an inhibition zone of 11.83, 17.33, and 9.00 mm, respectively.

*C. citratus* EOs disrupted the cell membrane and inhibited cytoplasmic metabolism, making them effective against both Gram-negative and Gram-positive bacteria.^3,104^ *Salmonella*, *Clostridium botulinum*, *E. coli*, *Campylobacter jejuni*, and *L. monocytogenes* were found to be susceptible to the effects of the EOs derived from the leaves of *C. citratus*, mainly β-citral (neral) and α-citral (geranial).^105^ The MIC and MBC of *C. citratus* EOs and their nanoemulsion forms against the bacteria tested are shown in Table 5. The bacteriostatic dose of pure *C. citratus* EO was 6.25 mg/mL against MRSA, *B. cereus*, and *P. mirabilis*. In contrast, the MIC against *S. Paratyphi* A. and *V. vulnificus* was 12.50 mg/mL. Placebo NE had 100 mg/mL of MIC, whereas NE1.5:1 (1% EO) and NE1.5:1 (5% EO) had a bacteriostatic dose of 50 and 12.5 mg/mL, respectively, against all bacteria tested apart from *P. mirabilis*. The MIC of *C. citratus* EOs ranged from 587.76 to 625.21 μg/mL, whereas the MBC was in the range of 1143–1250 μg/mL against test microorganisms.^106^ For all investigated bacteria, including Gram-positive (*B. cereus* and *S. aureus*) and Gram-negative (*E. coli* and *P. aeruginosa*) bacteria, the MIC values in the aqueous extract of lemongrass were comparable (6.25 μg/mL).^2^

**Table 5:** MIC and MBC of *C. citratus* EOs and Their Nanoemulsion Forms on Fish Spoilage Bacteria and Foodborne Pathogenic Bacteria (mg/mL)

		C. citratus EO	NE	NE1.5:1 (1% EO)	NE1.5:1 (5% EO)	NE1.5:1 (10% EO)	Tween 80	Cremophor	oleic acid
MRSA	MIC	6.25	100.00	50.00	25.00	12.50	>100	100	>100
	MBC	25.00	100.00	100.00	50.00	50.00	>100	>100	>100
S. Paratyphi A	MIC	12.50	100.00	50.00	25.00	25.00	>100	100.00	>100
	MBC	100.00	>100	>100	100	50.00	>100	>100	>100
B. cereus	MIC	6.25	100.00	50.00	25.00	12.50	>100	100.00	>100
	MBC	100.00	100.00	100.00	50.00	50.00	>100	>100	>100
V. vulnificus	MIC	12.50	100.00	50.00	25.00	12.50	>100	100.00	>100
	MBC	25.00	>100	50.00	25.00	25.00	>100	>100	>100
P. mirabilis	MIC	6.25	100.00	100.00	50.00	25.00	>100	100.00	>100
	MBC	50.00	100.00	100.00	100.00	25.00	>100	>100	>100

The MIC value in the NE1.5:1 (10% EO) group was found to be 12.5 mg/mL for most bacteria. Among the surfactants, the lowest MIC value was observed for 100 mg/mL Cremophor. Other surfactants did not exhibit antimicrobial activity at the concentrations tested (with MIC and MBC values of >100 mg/mL). Tween 80 did not influence microbial growth.^81^ Similar to this study, the surfactants (Tween 20, Tween 80, and Cremophor EL) showed no antibacterial activity with MIC and MBC values above 500 μg/mL.^107^

The lowest bactericidal dose of *C. citratus* EO (25 mg/mL) was observed against *S. Paratyphi* A and *V. vulnificus*. In contrast, the highest bactericidal dose of *C. citratus* EO (100 mg/mL) was against *B. cereus*. Following the *C. citratus* EO group, the most effective group in inhibiting bacterial growth was the nanoemulsion group with 10% and 5% *C. citratus* EO [NE1.5:1 (10% EO) and NE1.5:1 (5% EO) groups]. The MIC values of free and nanoencapsulated EOs did not differ.^98,108,109^ Similar results were observed only against *V. vulnificus* for free *C. citratus* EO and NE1.5:1 (10% EO). In general, the nanoemulsion forms at the tested EO doses displayed weaker bactericidal properties compared with their crude EOs. The NE1.5:1 (10% EO) dose had a lower MBC value against *S. Paratyphi* A, *B. cereus*, and *P. mirabilis*. Similarly, higher emulsion concentrations resulted in progressively better suppression of the growth of *S. typhimurium*, with 2× MIC reaching total inhibition according to the time-kill assay for *C. citratus* EO, exhibiting a dose-dependent impact.^99^ The MBC value in the NE1.5:1 (10% EO) group was 25.00 mg/mL against *V. vulnificus* and *P. mirabilis*. The NE and NE1.5:1 (1% EO) groups had similar MBC against bacteria (100 or >100 mg/mL) other than *V. vulnificus*, the NE1.5:1 (1% EO) group being more effective. *C. citratus* EO nanoemulsion formulations made with Tween 20 and Tween 80 showed complete inhibition against Gram-negative *E. coli* (MTCC-40) and *P. aeruginosa* (MTCC-424) and Gram-positive *B. subtilis* (MTCC-121).^110^ They also showed moderate antimicrobial activity against *S. aureus* (MTCC-3160). Daud et al. demonstrated concentration- and time-dependent impacts of *C. citratus* EOs and citral nanoemulsions on the growth of *B. cereus* isolates (ATCC 14579, P4, and M2).^111^ Both 0.5% and 2% *C. citratus* EOs or citral nanoemulsions almost completely inhibited the growth of *B. cereus* P4 and *B. cereus* M2 on day 0. *C. citratus* EO-based nanoemulsion showed an IC_50_ of 62 μg/mL against *S. aureus*, but it was much less potent against *P. aeruginosa* (5839 μg/mL).^102^

## 4 Conclusions

The EO analysis of *C. citratus* revealed a composition dominated by five major components: geraniol acetate, citral, β-citral, geraniol, and linalool. *C. citratus* EO-based nanoemulsions at three different concentrations (1%, 5%, and 10%) were evaluated in terms of droplet size, PDI, pH, conductivity, and viscosity. None of the concentrations tested (0.05 to 2 μg/mL) resulted in statistically significant reductions in cell viability after 24 h (*p* > 0.05). All of the tested formulations were found to be nontoxic because cell survival was more than 85% at the highest concentration of 2 μg/mL. Strong antibacterial action against both Gram-positive and Gram-negative foodborne pathogens was demonstrated by the EOs of *C. citratus*, with *S. Paratyphi* A being the most effectively targeted. Their nanoemulsions were more effective against MRSA and *B. cereus*. Despite improvements in delivery and stability by nanoemulsion formulations, their antibacterial activity was often weaker than that of pure EO. However, the antibacterial efficacy increased with increasing EO concentrations. Citral and geraniol, two essential components that interfere with bacterial membranes and metabolism, were identified as contributors to the antibacterial effect. These findings suggested the use of *C. citratus* EOs and their nanoemulsions as natural resources to produce potent antibacterial agents for applications in food and medicine.
